# Immunological and Histological Studies of Different Concentrations of *Rosmarinus officinalis* and *Thymus vulgaris* Extracts on Thymus Gland of Chick Embryos

**DOI:** 10.3390/toxics11070625

**Published:** 2023-07-19

**Authors:** Reem Yahya Alzahri, Fawzyah Abdullah Al-Ghamdi, Seetah Saleem Al-Harbi

**Affiliations:** Department of Biology, College of Science, University of Jeddah, Jeddah 21493, Saudi Arabia; faalgamdi@uj.edu.sa (F.A.A.-G.); salharbi0902.stu@uj.edu.sa (S.S.A.-H.)

**Keywords:** embryo developments, thymus gland, IgM, IgG, IL-10, rosemary, thyme

## Abstract

Humanity has an ancient history of consuming medicinal plants for prophylaxis. Within hours, and even months, embryonic cells undergo several processes to form an organism. This study aimed to prove the positive or negative effects of using rosemary and thyme extract on the thymus gland and level of IL-10, IgM, and IgG in serum of chick embryos. The immunological effect was measured by histological and immunological studies. A total of 160 fertilized eggs were randomly distributed into 8 groups; on the 0 and 8th day of incubation, all treated groups received a dose of 0.1 mL/egg. On the 14th and 20th days of incubation, the embryos were sacrificed and the samples were collected (serum and thymus gland). The data were analyzed using ANOVA. Simple damage in thymic tissue with a low cell density in the embryos was treated with high concentrations of rosemary and thyme extracts, as well as in the mixed group. A significant decrease in IgM levels in the group treated by a high concentration of thyme. A decrease in IgG levels was found in the group treated with a high concentration of rosemary and in the mixed group, while the group treated with a high concentration of thyme and the mixed group showed decreases on the 14th day. A significant decrease in IL-10 levels was found on the 14th day, followed by an increase on the 20th day. Despite the benefits of rosemary and thyme, inflammation signs appeared on embryos treated with these herbs.

## 1. Introduction

The immune system orchestrates a body via group of cells and soluble molecules to provide protection and homeostasis; this system consist of components including organs such as the thymus gland, cells, and soluble molecules such as cytokines and antibodies [1]. The thymus gland is the primary organ in the immune system, which secretes several hormones with important roles in the growth and function of lymphatic tissue, such as thymosin [2,3]. The thymus is a bi-lobed organ in the thoracic cavity of mammals and a series of lobes in the neck of birds and reptiles [4]. In humans, the thymus is a bi-lobed mass located behind the sternum [5]. It is a paired lobulated organ, translucent, bean-shaped and consisting of 6–8 lobes that adhere with each other through connective tissue in the chicken. The thymus is located in both sides of the neck, positioned parallel to the jugular vein and vagus nerve [6]. T-cells are the main lymphoid cell type in the thymus gland, in addition to thymic epithelial cells [7]. Furthermore, it contains other types of cells, such as macrophages and dendritic cells. The thymus consists of two distinct parts; the peripheral part of the organ is called the cortex while the central part is called the medulla. The lobes are surrounded by connective tissue called capsule: a loose connective tissue extends from the capsule to the cortex to divide it into lobules. The capsule is an essential part of the thymus and provides support to the organ [8]. The cortex is the peripheral part of the gland and is darkly stained and densely packed compared to different cells, while the medulla is the central part of thymus, containing mature T lymphocyte and a high density of epithelial cells with pale staining nuclei [9].

The soluble molecules play a dominant role in the functionality of the immune system [10]. Antibodies (or immunoglobulin) are a unique type of proteins found in blood and body fluids of vertebrates [11]. They are secreted by B-lymphocytes after differentiation into plasma cells. There are five types of immunoglobulins: IgA, IgD, IgG, IgE, and IgM. Immunoglobulin G (IgG) antibodies are large monomeric globular proteins and represent from approximately 70% to 75% of the total antibodies in plasma. IgG is capable of transporting across the placenta to the embryo [12]. IgG levels increase in patients with cancer and hepatitis [13]. IgG decreases in some cases, such as in children diagnosed with a specific allergy [14]. Immunoglobulin M (IgM) is the first antibody to appear after an infection or immunization, and is the first antibody an embryo makes [15]. IgM is not capable of transporting through the placenta to embryo; therefore, its presence in the umbilical cord blood is an indication of a prenatal infection [16]. To maintain homeostasis in multicellular organisms, communication is required between cells. One of the most important secretion for cell communication is through the cytokines [17]. Interleukins (IL) are a type of cytokine that were once considered to be produced only by leukocytes, but recently have been discovered to be produced by different types of cells; they are involved in immune cell activation and differentiation and also showed an anti-inflammatory and pro-inflammatory effects [18]. IL-10 is a powerful anti-inflammatory cytokine with potent anti-inflammatory properties that suppresses the expression of inflammatory cytokines such as TNF-α, IL-6, and IL-1 by inhibiting macrophages, which is responsible for the production of pro-inflammatory cytokines [19,20].

Recently, the use of medicinal plants has significantly increased, specifically due to their having a high nutritional value [21]. The prevalence of using medicinal plants compared to chemical drugs is due to many reasons: their accessibility, the belief that they gave no side effects and can cure different diseases, and their ability to boost immunity [22].

Medicinal plants have been increasingly used in many countries. Women around the world use medicinal plants, especially in the first trimester of pregnancy [23]. Some pregnant women prefer to use medicinal plants as a remedy due to the effortless accessibility to herbs and the notion that herbs have no side effects [24]. The risks of using medicinal plants increase in some cases, such as in pregnancy, and most medicinal plants are not supported by safety studies [25]. Consuming a specific medicinal plant during the first trimester of pregnancy may increase the risk of congenital defects [26]. Of these medicinal plants, two plants are commonly used in our lives: *Rosmarinus officinalis* (rosemary) and *Thymus vulgaris* (thyme). Rosemary and thyme are medicinal plants belonging to the lamiaceae family, which is rich in active ingredients. They have many advantages, for example, antimicrobial and antioxidant properties and the ability to improve immunity [27]. Thyme and rosemary are evergreen herbs and have an aromatic scent. Rosemary extract is used for several purposes, including therapy and cooking, and is also used in aromatic perfumes. Rosemary oil is widely used in cosmetic products and is currently being used as a treatment to stimulate hair growth [28]. Thyme is popular as a hot drink; in addition to this, it can be added to dishes and used as a remedy. Thyme extract and its oil are distinguished by their use as an antiseptic; moreover, they are used in cosmetics [29,30]. Rosemary is rich in chemical compounds such as apigenin, diosmin, luteolin, rosmarinic acid, caffeic acid, camphor, α-pinene and borneol [31]. In parallel, thyme is rich in flavonoids, thymol, carvacrol, borneol, eugenol, phenols, luteolin, camphene, camphor and tetra methoxylated [32,33].

As mentioned earlier, rosemary and thyme are rich in active ingredients [34]; therefore, they can cause damage when accumulating in an organism’s body. The essential oils in some plants might be acutely toxic in small amounts [35]. As example, exposure to low levels of essential oils of the thyme plant (thymol and carvacrol) showed toxicity in embryonic growth [36]. According to the Physician’s Desk Reference for Herbal Medicines, rosemary should not be used as a drug during pregnancy, whereas high quantities can be misused as an abortifacient [37]. Allergy to members of the Lamiaceae family or its ingredients may cause asthma and dermatitis [38]. Thyme oil, when taken internally, may cause vomiting, and high-blood-pressure patients must avoid it [39].

Among all living organisms, embryos have a high sensitivity to any foreign matter that enters their environment. On the other hand, the immune system of embryo is responsible for defense and recognizing any foreign substances. In addition, the developing embryo creates a mechanism of defense via equilibrium between itself and the maternal immunological system.

Accordingly, in case of an inability to regulate the substances that enter the embryonic environment, the immune defense mechanisms will fail in terms of embryo protection. The embryo lives in a suitable environment and requires pivotal material such as gases and nutrition to grow and develop; however, certain quantities are required to prevent the reflection of beneficial factors that may cause adverse effects. Deficiencies or increases in the materials supplied to the embryo lead to deviations in aspects of embryo development such as nutrition [40]. Despite the nutritional value of rosemary and thyme, there are no sufficient studies on their effect, especially their effect on the immune system during embryogenesis. For this reason, this study aimed to show the effect of using rosemary and thyme in different concentrations on the thymus gland and the level of IgM, IgG antibodies, and IL-10 cytokine in the serum of chick embryos.

## 2. Materials and Methods

### 2.1. Test Reagents, Chemicals, and Materials

The chemical and reagents were obtained from Sigma-Aldrich and the chicken IgM, IgG, and IL-10 ELISA Kit was purchased from (Bioassay Technology Laboratory, Jiaxing, Zhejiang Province, China), from the following link (http://www.bt-laboratory.com accessed on 8 April 2022). Rosemary and thyme dry leaves were obtained from local herb markets in Jeddah, Saudi Arabia.

### 2.2. Animals and Incubation

Fertilized eggs were obtained from poultry farms in Jeddah, Saudi Arabia. A total of 160 fertilized eggs were used in the current study. The average egg weight was (~60 g), and the eggs were normally incubated at 37.7 °C and 65% relative humidity [41] in an automatic incubator.

### 2.3. Dose Preparation and Administration

Eight grams of rosemary powder and 10 g of thyme powder were dissolved in 100 mL of boiling water, separately for each herb; then, they were soaked for 10 min before filtration using sterile filter paper (Grade 2, 90 mm diameter) [42,43]. Several concentrations of plant extract were created using the dilution equation (V1C1 = V2C2). High (100%), medium (50%) and low (10%) concentrations were prepared from rosemary and thyme extracts. Moreover, we prepared a dose by mixing these two herbs at 50% concentration. The dose injected for all treated groups was 0.1 mL/egg, according to [41], for thyme extract; depending on the dose converted from the study of [44], the rosemary extract injection contained 0.1 mL/egg.

### 2.4. Experimental Design

After preparing the plant extracts, 160 fertilized eggs were randomly divided into 8 groups (n = 20). These groups included a control group that received saline solution (A1), and the treatment groups, which were rosemary high con. (100%—B1), rosemary medium con. (50%—B2), rosemary low con. (10%—B3), thyme high con. (100%—C1), thyme medium con. (50%—C2), thyme low con. (10%—C3) and mixed group (rosemary medium con. (50%) + thyme medium con. (50%—D1). All of these groups were injected into the air sac according to the procedure described by [45]. The injection occurred on day 0, the time before incubation, and this day is considered as a day of the sensitive period; furthermore, another injection occurred on the 8th day in the second week of the incubation period, in which the embryo has developed all its organs and will gradually begin to grow.

### 2.5. Histological Study of the Thymus Gland

The thymus gland was obtained on the 14th and 20th days (at the end of the “second–third” week) of incubation and fixed in 10% neutral buffered formalin to prepare for Eosin and hematoxylin stains (using the routine histological technique). The stained section will be examined by virtual microscope systems (online access to pathology slide scanner (Version 3.2, Philips & Co., Amsterdam, The Netherlands, 2023) at several magnification powers).

### 2.6. Immunological Study of the Serum

The blood samples were collected on the 14th and 20th days (at the end of the “second –third” week) of incubation. Blood samples were collected from jugular veins and umbilical veins in a plain centrifuge tube and the blood coagulated at room temperature for approximately 1 h before separating. The serum was separated and centrifugation occurred at 4000 rpm for 15 min; after centrifugation, the serum was carefully separated from the resulting supernatant; then, the serum was put in plastic tubes and stored in a freezer at −20 °C until analysis [46]. Immunological studies were performed by enzyme-linked immunoassay (ELISA) for IL-10 as anti-inflammatory cytokines and IgG and IgM antibodies. In the ELISA test, we followed the steps in the instruction paper. The absorbance was read in each well using a microplate reader and the absorbance was recorded at 450 nm. According to standard concentrations and the corresponding absorbance values, the linear regression equation of the standard curve was calculated (y = mx + b).

### 2.7. Statical Analysis

The data were analyzed using one-way analysis of variance (ANOVA) in the SPSS program (version 22) and represented graphically using GraphPad Prism (version 9.4.0).

## 3. Results

Using histological and immunological studies, the results were obtained; the key aspects of the results will be described in the following.

### 3.1. Microscopic Examination of Thymic Tissue

**Figure 1:**
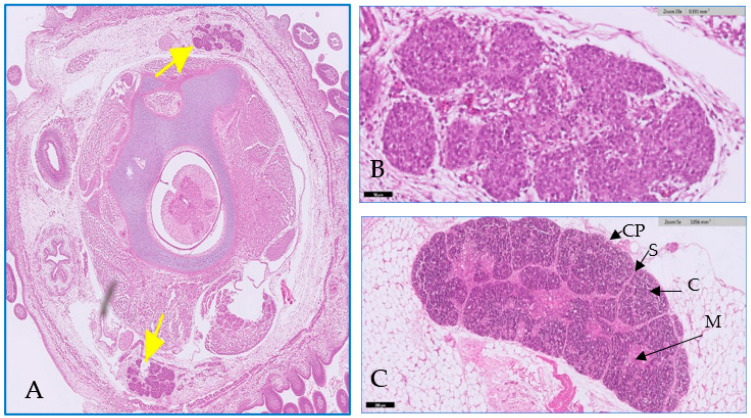
Showing the growth in the thymus gland over time. In Figure (**A**,**B**) the thymus appears during the second week of incubation (14th day), and in Figure (**C**), in the last week of incubation (20th day), an accurate structure of the thymus gland appears; which was encapsulated by a capsule (CP) and the lobules separated by septa (S); inside the gland, the cells organized and distributed in the cortex © and medulla (M). H&E stain. Scale bar was 1 mm, 50 µm, and 200 µm, respectively.

**Figure 2:**
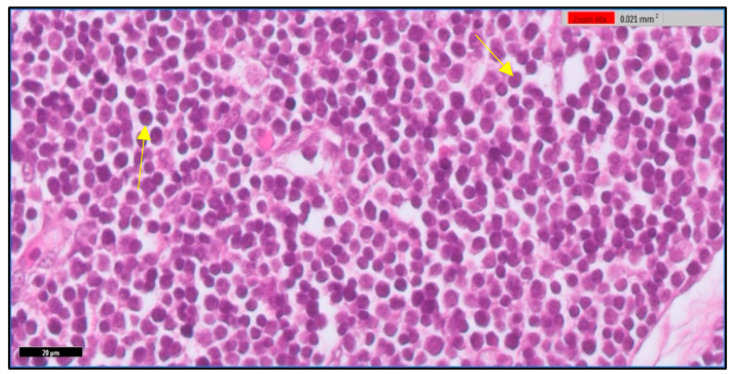
Shows the cortex of the thymus gland. It is darkly stained and abundant numbers of lymphocytes appear (yellow arrow). H&E stain. Scale bar 20 µm.

**Figure 3:**
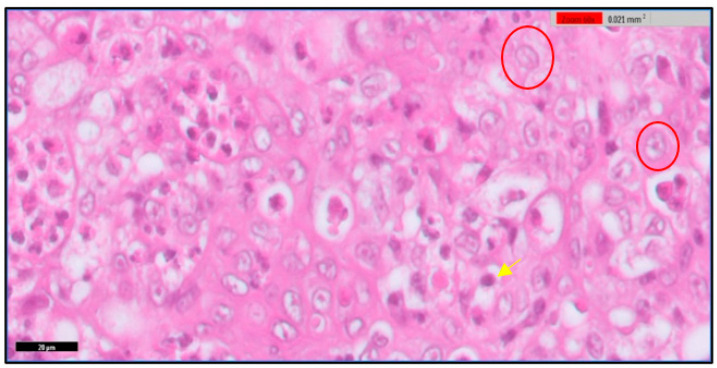
Shows the medulla of the thymus gland. There are several mature lymphocytes (yellow arrow) and a high density of epithelial cells with pale stained nuclei (red circle). H&E stain. Scale bar 20 µm.

**Figure 4:**
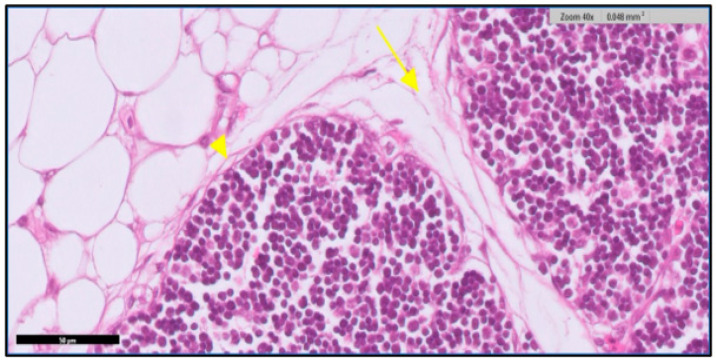
Shows the capsule and septa of the thymus gland. A part of the connective tissue of the capsule (arrowhead) invaginates the thymus to form the septae (arrow). The thymus appears surrounded by adipose tissue. H&E stain. Scale bar 50 µm.

The thymus gland is in a parallel position to the jugular vein and vagus nerve on the right and left side of the neck, as shown in Figure 1A. It consists of two main regions and will become more differentiated with an increase in embryo age, as shown in Figure 1 (B&C). The first is the cortex, which is the peripheral part of the gland and is denoted by dark staining, as shown in Figure 2. The central region is the medulla; in this part, there are several mature T lymphocytes and a high density of epithelial cells with pale staining nuclei, as shown in Figure 3. The thymus gland is covered by a connective tissue capsule, and the capsule tissue invaginates the thymus to form the septa, as shown in Figure 4.

#### 3.1.1. At 14th Day

**Figure 5:**
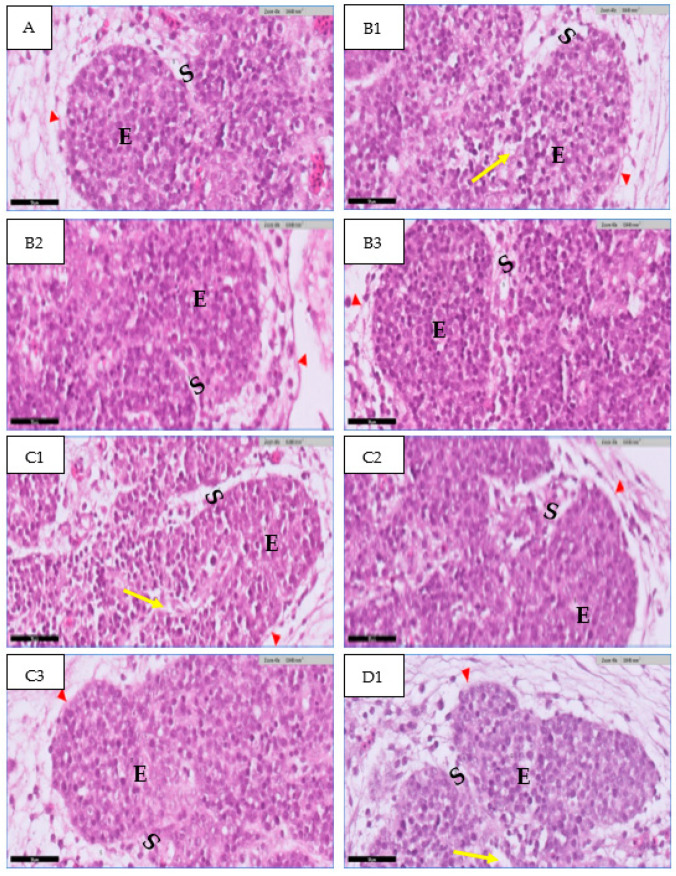
Photomicrographs of a section in the thymus gland of a chick embryo at the 14th day of incubation. A: (control group) shows the lobules of the thymus consisting of epithelial cell mass (E) with a low number of lymphocytes surrounded by basement membrane (Red arrowhead) and the lobules separated by the interlobular mesenchyme(septa) (S). In the group treated with a high concentration of extract and in the mixed group, degradation and spacing can be seen between cells, as well as a reduction in cell density (Yellow arrow). (H&E) stain. Scale bar 50 µm.

In the present study, the histological examination of the thymus section at the 14th day showed the normal structure of the thymus in the control group. The thymus appears in the lobules’ structure. There are many cells in the thymus, such as a large cell with round or oval nuclei called the epithelial cell, which is found in large amounts, and another type of cell that is found in small amounts, which has darkly stained nuclei with a small cell called a lymphocyte. The cell’s mass is surrounded by connective tissue called the basement membrane; this connective tissue extends deep into the center of the lobe to form the septa (Figure 5A). A low density of epithelial cells and lymphocytes was found in the group treated with 0.1 mL of high-concentration rosemary extract (Figure 5B1). In the group treated with high-concentration thyme extract, atrophic changes were found (low cell density) with high spacing between cells (Figure 5C1). A normal structure of thymus tissue and normal cell density appeared in the groups treated with 0.1 mL of rosemary and a medium thyme concentration when compared to the control group (Figure 5B2,C2). A normal structure of thymus tissue appeared in the group treated with 0.1 mL of low-concentration rosemary when compared to the control group (Figure 5B3). The researchers did not notice any abnormal change in thymus tissue in the group treated with 0.1 mL of low-concentration thyme (Figure 5C3). As seen in Figure 5; D1, the thymus tissue in the mixed group appeared, with a low cell density for the epithelial cell and lymphocyte.

#### 3.1.2. At 20th Day

**Figure 6:**
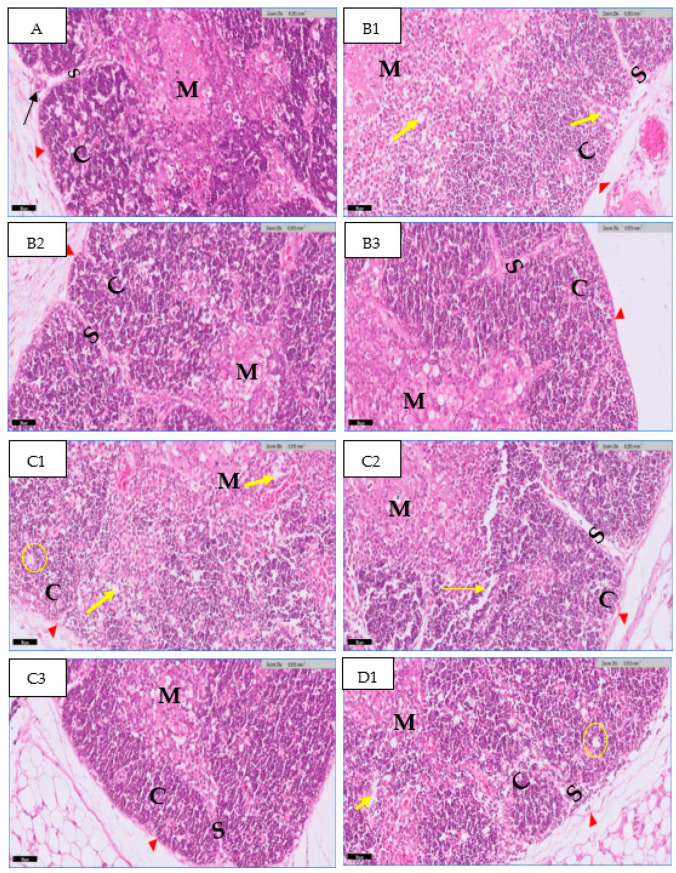
Photomicrographs of a section in the thymus gland of chick embryo at 20th day of incubation. A: (control group) shows the lobules of the thymus, consisting of two regions, the cortex (C) and medulla (M), enclosed by a capsule (Red arrowhead); the lobules separated by septa (S) and the blood vessels also appear (black arrow). In the group treated with a high concentration of extracts and a mixed group, degradation and spacing could be seen between cells, as well as a reduction in cell density (Yellow arrow), and vacuoles (circle). (H&E) stain. Scale bar 50 µm.

Histological examination of the embryonic thymus section at the 20th day of incubation showed the normal structure of the thymus in the control group at the 20th day of incubation; the thymus increased in size and appeared to be surrounded by thin capsules. Lobules are separated by connective tissue and become more vascular. The number of lymphocytes in the thymus tissue increased when compared to the previous age of gland development. The cortex mainly consists of immature lymphocytes (darkly stained) and a low amount of epithelial cells. The medulla area consists of a high population of epithelial cells with few lymphocytes (mature lymphocytes) (Figure 6A1).

In the groups treated with 0.1 mL of high-concentration rosemary, the thymus section showed a low cell density in the cortex and medulla (Figure 6B1). In the group treated with high-concentration thyme, a low cell density (atrophic changes) was found in the cortex and medulla, and the cells appeared to be distributed in the cortex of the thymus (Figure 6C1). The groups treated with 0.1 mL of medium-concentration rosemary showed a normal appearance of the thymus, with a cellular density resembling the control group (Figure 6B2). The group treated with medium-concentration thyme showed a partial decrease in cell density with spacing between cells (Figure 6C2). The groups treated with 0.1 mL of low-concentration rosemary had a normal appearance of the thymus with a cellular density similar to the control group (Figure 6B3), whereas the group treated with a low concentration of thyme showed normal cellular density with a normal appearance, similar to the control group (Figure 6C3). The mixed group showed an abnormal thymus appearance and low cell density compared to the control group with vacuoles in tissue and cell destruction (Figure 6D1).

### 3.2. Immunodiagnostics of Antibodies and Cytokine in Serum

#### 3.2.1. Cytokine Production upon Treatment with Rosemary and Thyme Extract

**Figure 7:**
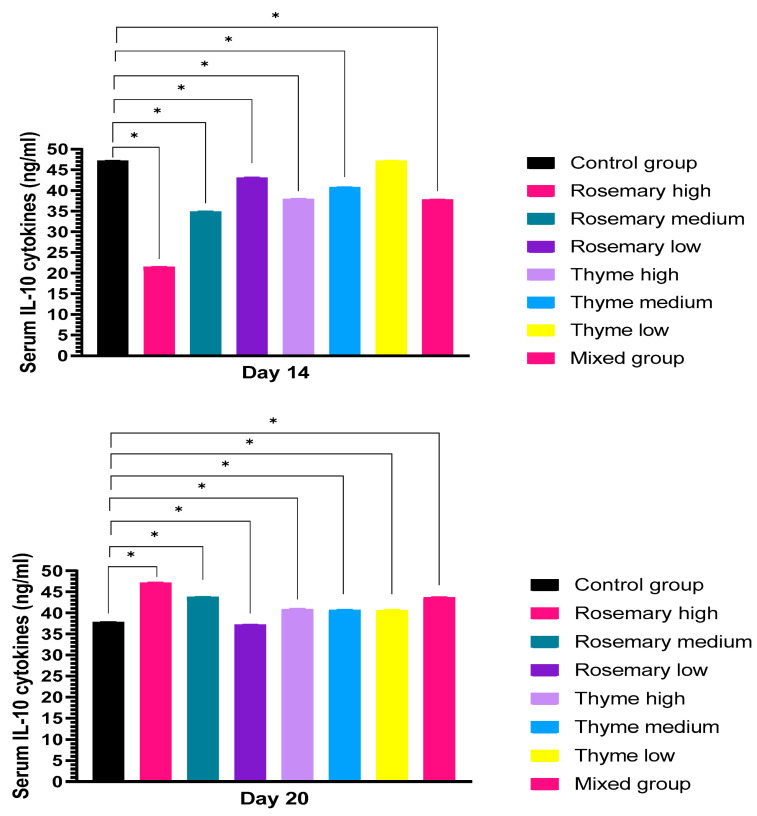
Shows the level of IL-10 cytokine production that was determined in the serum of chick embryo using ELISA kits after embryo exposure to two doses of rosemary and thyme extracts in different concentrations with a dose of (0.1 mL). The data shown in the figure represent the mean ± SD (n = 4), and there is a significant difference between groups at a significance level of *p* < 0.05 *. At day 14, there are significant decreases in all groups exceptthe low-concentration thyme group. After three weeks of incubation and exposure to two doses of rosemary and thyme extract, a significant increase was recorded in all groups except the group treated with low-concentration rosemary.

The level of (IL-10) production at day 14 of incubation showed a decrease in both groups treated with rosemary and thyme extract compared with the control group, by (47.2 ng/L). The level of IL-10 in the rosemary-treated group, for high, medium and low concentrations, was 21.6 ng/L, 34.9 ng/L and 43.3 ng/L, respectively. The cytokine level in the thyme-treated group for high and medium concentrations was 37.9 ng/L and 40.8 ng/L, respectively. Low-concentration thyme was not shown to induce cytokine production (47.2 ng/L). In the mixed group, there is a significant reduction of IL-10 in the serum by (37.8 ng/L). At day 20 of incubation, the level of cytokine production increased in all treated groups compared to the control group by (37.8 ng/L), except in the group treated by low-concentration, which showed a similar value to the control group. The cytokine level in the group treated with rosemary extract was 47.2 ng/L, 43.9 ng/L, and 37.2 ng/L, from high to low concentration, respectively, and 40.9 ng/L, 40.7 ng/L, and 40.6 ng/L for high, medium, and low concentrations of thyme extract. The mixed group showed a significant increase when compared to the control group, by 43.7 ng/L, as shown in Figure 7.

#### 3.2.2. Antibody Production upon Treatment with Rosemary and Thyme Extract

##### IgM

**Figure 8:**
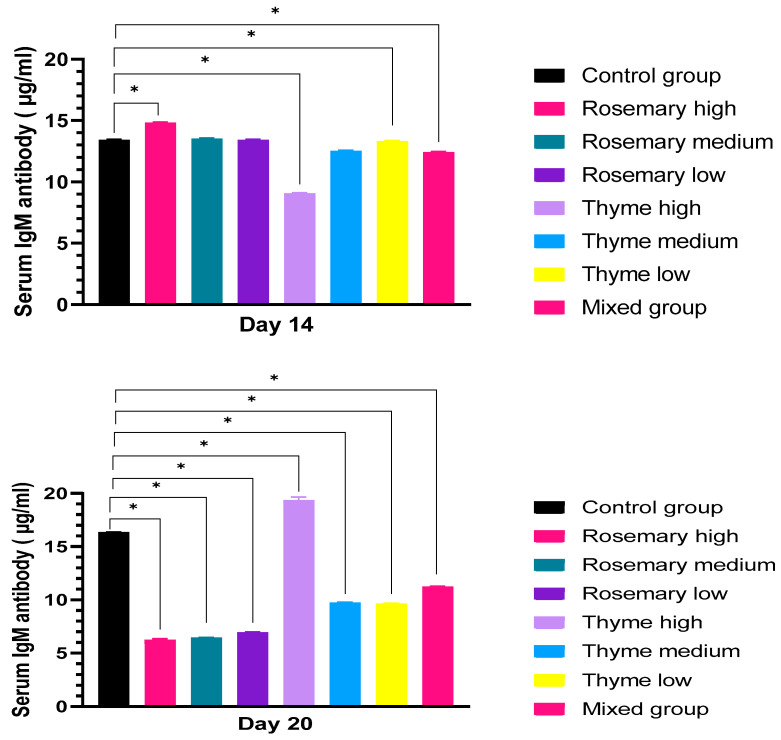
Shows the level of IgM antibody production determined in the chick embryo serum using ELISA kits after embryo exposure to two doses of rosemary and thyme extracts in different concentrations, with a dose of 0.1 mL. The data shown in figure represent the mean ± SD (n = 4), and there is a significant difference between groups at the level of significance (*p* < 0.05) *. After two weeks of incubation, there was a significant decrease in high and medium concentrations of thyme and the mixed group, and a significant increase in the high-concentration rosemary group. At day 20, and after three weeks of incubation and exposure to two doses of rosemary and thyme extract, the IgM antibodies secretion was significantly decreased in all groups, except in the high-concentration thyme group, where there was an increase in antibody production.

At the 14th day of incubation, IgM antibodies showed an increase in the rosemary-treated group with a high and medium concentration, by 14.8 ug/mL and 13.5 ug/mL, while low-concentration rosemary showed an equivalent value for the control group by 13.4 ug/mL. In the groups treated with thyme, high, medium and low concentrations showed a reduction in IgM antibody level by 9 ug/mL, 12.5 ug/mL, and 13.3 ug/mL. IgM antibody level decreased in the mixed group by (12.4 ug/mL). At 20th day of incubation, IgM production decreased in all rosemary groups by 6.2 ug/mL, 6.4 ug/mL, and 6.9 ug/mL respectively from high to low concentrations compared to the control group, which showed a decrease of 16.3 ug/mL. A high concentration of thyme induced the production of IgM by 19.3 ug/mL, while IgM antibody production decreased with medium and low concentrations of thyme, by 9.7 ug/mL and 9.6 ug/mL. In the mixed group, the IgM level production decreased by 11.2 ug/mL, as shown in Figure 8.

##### IgG

**Figure 9:**
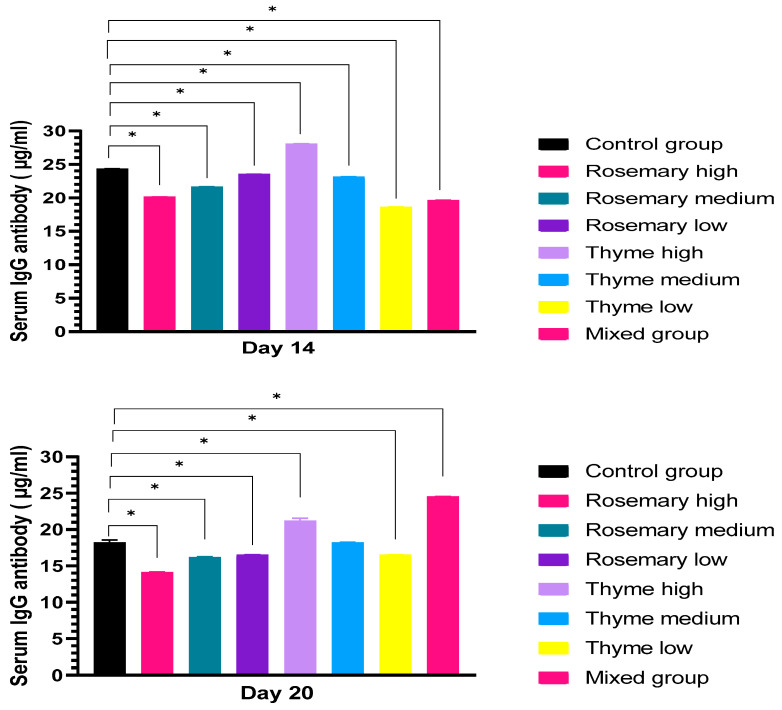
Shows the level of IgG antibody production that was determined in the serum of chick embryo using ELISA kits after embryo exposure to two doses of rosemary and thyme extracts in different concentrations, and the dose was 0.1 mL. The data shown in the figure represent the mean ± SD (n = 4), and there is a significant difference between groups at the level of significance (*p* < 0.05) *. At day 14 and after two weeks of incubation, there was a significant decrease in all groups and a significant increase in high-concentration thyme. At day 20 and after three weeks of incubation and exposure to two doses of rosemary and thyme extract, there was a significant increase in antibody secretion in the group treated by high-concentration thyme and the mixed group, while a decrease was seen in all other groups except medium-concentration thyme.

IgG antibody level at the 14th day of incubation showed a decrease in rosemary for high, medium, and low concentrations (20.1 ug/mL, 21.6 ug/mL, and 23.5 ug/mL, respectively) compared to the control group (24.4 ug/mL). IgG antibody levels showed an increase in the high-concentration thyme group by 28 ug/mL, and a decrease in medium- and low-concentration thyme by 23.1 ug/mL, and 18.6 ug/mL. The level of IgG production decreased in the mixed group by 19.6 ug/mL. On the 20th day of incubation, in the groups treated with high-, medium-, and low-concentration rosemary, the results showed a reduction in IgG antibody level by 14.1 ug/mL, 16.2 ug/mL and 16.5 ug/mL, respectively, compared to the control group (18.2 ug/mL). IgG antibody level increased in high-concentration thyme by 21.2 ug/mL and the medium-concentration thyme group does not induce cytokine production (18.2 ug/mL), while a decrease was seen in low-concentration thyme by 16.6 ug/mL. In the mixed group, the level of IgG production increased by 24.3 ug/mL compared to the control group, as shown in Figure 9.

## 4. Discussion

The immune system comprises a complex network of cells and soluble molecules, which collectively have a protective role against infections, tumors and maintain homeostasis after inflammation [47,48]. The thymus gland is a lymphoepithelial organ that belongs to primary lymphoid organs in the immune system [49]. Medicinal plants have been used to cure diseases and boost immunity [22]. It is known that they are involved in different applications that can modify the immune system either positively or negatively [50]. In this work, we investigate the impact of different doses of plant extracts (rosemary and thyme); we studied different measurements after exposing chick embryos to these extracts. The data were generated by using a microscopic examination of thymic tissue, in addition to the immunodiagnostics of antibodies (IgM-IgG) and cytokine (IL-10) in serum. Surprisingly, rosemary and thyme extracts are known to be rich in antioxidants, with a harmful effect on embryos.

In the thymic tissue, the epithelial cell mass in the gland appeared, with a low density in the groups treated by high concentrations of rosemary and thyme and in the mixed group after 14 days of incubation. Furthermore, a significant decrease was experienced in lymphocyte density in the thymus gland at the 20th day of incubation. The thymus gland is the target organ for growth hormones [51] and the body usually needs high levels of growth hormones to activate the thymus gland, to promote T cell maturation during the early stages of development [52]. However, it is obvious that injection with high doses of extract in the mixed group led atrophic changes with low cell density in thymic tissue, and this atrophy usually occurs in old age when the growth hormone secretion is reduced [53]. It is conceivable that the high-concentration rosemary and thyme extracts have a high amount of active ingredients, and this perhaps led to a harmful effect. One of the major chemotypes in rosemary and thyme is a camphor; a study by [54] showed the toxicity effect of camphor on rat embryos and atrophic changes in the uterus of pregnant rats. Deficiencies in the lymphocytes and the abnormal development of the thymus gland might indicate a defect in the third and fourth pharyngeal pouches during embryo development [55].

The level of IL-10 in serum is a hallmark value for prognosis regarding oxidative stress. At the 14th day of incubation, the IL-10 level experienced a significant decrease and this reduction is commonly caused by the disruption of the immune system in the case of inflammation, or infection; for example, a reduction in IL-10 level is an indication of inflammation and severe immune system response [56]. IL-10 levels were significantly reduced in the embryo serum in the group treated by different concentrations of thyme and rosemary extracts at the 14th day of incubation. On the other hand, IL-10 levels significantly increased before hatching at the 20th day of incubation. We speculate that the proinflammatory cytokine response was predominant in the embryos after a week of receiving two doses. On the contrary, the level of IL-10 on the 20th day of incubation was a positive indicator for a decline in inflammation. To illustrate this, the increase in IL-10 levels in the serum lead to inhibition of inflammatory response, and the influence of inflammatory factors will decrease [57]. Consistent with previous studies, the rosemary and thyme led to an increase in IL-10 levels. A similar study was conducted by [58], when incubated human macrophages were derived from the human leukemia monocytic cell line (THP-1) by thyme extract oil; this agreed with the positive results of [59] that treating adult male rats with thyme oil, after exposure to carbon tetrachloride (CCl4), caused toxicity [60]. The current results substantially agreed with the previous investigation of [61], who studied the anti-inflammatory activity of *Rosmarinus officinalis* plant in a systematic review for several previous studies that showed an increase in anti-inflammatory biomarkers, for example, an increase in IL-10 level in mouse models with Pleurisy, which is induced by carrageenan after the administration of *Rosmarinus officinalis* [62]. 

During embryogenesis, the living organism receives antibodies from the placenta or yolk sac. Based on previous research, IgG is the only antibody that can transport from maternal blood into the embryo [63]. The immunoglobulin level in serum determines the health state of the organism. If this level is high, it is an indication of allergies, inflammation and autoimmune disorder [64,65,66]. A severe decrease in immunoglobulins may indicate failure of B cell formation or defects in the bursa of Fabricius, which is responsible for B cell formation in birds [67,68]. In the same way, the low level of immunoglobulin in serum indicates a weak immune system, which may occur following the consumption of medicine such as non-steroidal anti-inflammatory drugs [69,70]. Moreover, variations in the level of antibody concentration in the serum of chick embryos may be attributed to gender differences; a previous study on a group of children announced that the girls had a higher level of IgG and IgM than boys [71]. When treating the embryos with thyme extracts, the IgG and IgM levels significantly increase, and this result is in agreement with several previous studies; for example, in Ovo, a 0.1 mL injection of thyme extract at day 10 of incubation [41] increased the level of IgC and IgM in serum; according to [72], treating the mice with (0.5 g/kg) of thyme extract had the same effect. In the group treated by high-concentration rosemary, the IgM secretion increased, and this result agreed with several previous results. For example, the level of IgM antibody increased when treating the mice with rosemary extract; furthermore, with the administration of rosemary oil in Ovo at the 18th day of incubation, the level of IgM secretion increased [73,74]. On the other hand, the reduction in IgM may contribute to an increase in the apoptotic cells. IgM plays an important role promoting macrophages clearance for small molecules, for example, apoptotic cells (2–5 micron) and apoptotic microparticles (<2 µm) [75]. The level of IgG in serum decreased in some experimental groups in the current study; for example, it decreased in the group treated with a high concentration of rosemary extract. The reduction in IgG indicates several problems; for instance, IgG deficiency increases the risk and mortality rate in chronic obstructive pulmonary disease [76]. According to a study by [77], the reduction in IgG level in laboratory diagnosis is associated with infections in children.

## 5. Conclusions

Rosemary and thyme are medicinal plants that have an important role in promoting health and improving immunity; however, overconsumption should be avoided during pregnancy because of the potential harmful effects to the embryo. This research concludes that consuming separated/mixed extracts of rosemary and thyme has an immunological adverse effect on embryos treated by high-concentration extracts; for example, reduction in the cell density of thymic tissue, reductions in the level of IgM and IgG in the serum of some groups, and a reduction in IL-10 cytokine. All these are a sign of inflammation, which is a dominant mechanism that the body uses to defend against injury or infection, which may lead to irreversible effects when left unchecked, and this can cause abnormal embryo development.

## Data Availability

Not applicable.
